# Inland valley rice production systems and malaria infection and disease in the forest region of western Côte d’Ivoire

**DOI:** 10.1186/1475-2875-12-233

**Published:** 2013-07-10

**Authors:** Serge-Brice Assi, Marie-Claire Henry, Christophe Rogier, Joël Dossou-Yovo, Martine Audibert, Jacky Mathonnat, Thomas Teuscher, Pierre Carnevale

**Affiliations:** 1 Institut Pierre Richet (IPR) de Bouaké/Institut National de Santé Publique (INSP), BP V 47, Abidjan, Côte d’Ivoire; 2 Institut Pasteur de Madagascar, B.P. 1274, Ambohitrakely, 101, Antananarivo, Madagascar; 3 CNRS, Centre d’Etudes et de Recherches sur le Développement International (CERDI), Université d’Auvergne, 65 Boulevard François Mitterrand, 63000, Clermont-Ferrand, France; 4 West Africa Rice Development Association (WARDA) currently known as the Africa Rice Centre, 01 BP 2551, Bouaké 01, Côte d’Ivoire

**Keywords:** Malaria, Disease, Infection, Rice cultivation systems, Forest, Côte d’Ivoire

## Abstract

**Background:**

This study aimed to determine the epidemiological impact of rice cultivation in inland valleys on malaria in the forest region of western Côte d’Ivoire. The importance of malaria was compared in terms of prevalence and parasite density of infections and also in terms of clinical malaria incidence between three agro-ecosystems: (i) uncultivated inland valleys, (R0), (ii) inland valleys with one annual rice cultivation in the rainy season, (R1) and (iii) developed inland valleys with two annual rice cultivation cycles, (R2).

**Methods:**

Between May 1998 and March 1999, seven villages of each agro-ecosystem (R0, R1 and R2) were randomly selected among villages pooled by farming system. In these 21 villages, a total of 1,900 people of all age groups were randomly selected and clinically monitored during one year. Clinical and parasitological information was obtained by active case detection of malaria episodes carried out during eight periods of five consecutive days scheduled at six weekly intervals and by cross-sectional surveys.

**Results:**

*Plasmodium falciparum* was the principal parasite observed in the three agro-ecosystems. A level of holoendemicity of malaria was observed in the three agro-ecosystems with more than 75% of children less than 12 months old infected. Geometric mean parasite density in asymptomatic persons varied between 180 and 206 *P. falciparum* asexual forms per μL of blood and was associated with season and with age, but not with farming system. The mean annual malaria incidence rate reached 0.7 (95% IC 0.5-0.9) malaria episodes per person in R0, 0.7 (95% IC 0.6-0.9) in R1 and 0.6 (95% IC 0.5-0.7) in R2. The burden of malaria was the highest among children under two years of age, with at least four attacks by person-year. Then malaria incidence decreased by half in the two to four-year age group. From the age of five years, the incidence was lower than one attack by person-year. Malaria incidence varied with season with more cases in the rainy season than in the dry season but not with farming system.

**Conclusion:**

In the forest area of western Côte d’Ivoire, inland valley rice cultivation was not significantly associated with malaria burden.

## Background

Africa has the highest population growth rate of any continent [1]. This explosive rate of population growth requires a great increase in food production. The severe pressure to feed the growing population has lead many African governments to look for better methods of expanding agriculture production, such as irrigation. Irrigation may be the most effective way to increase crop production through increased yield, acreage, number of cropping cycles per year, and by reducing the risk of crop failure [2]. However, in sub-Saharan Africa, water resource development projects affect many waterborne diseases. In the case of malaria, irrigated rice cultivation favours the development of *Anopheles gambiae s.l*. populations. Furthermore, the epidemiological impact of rice cultivation varies according to the local malaria situation [3]. A review of the literature shows that high vector density does not necessarily imply an increased risk of exposure to malaria parasite. In some areas, it can be associated with an increase in malaria transmission and morbidity, such as in Burundi and the highlands in Madagascar [4,5]. Conversely, irrigated rice cultivation does not seem to affect malaria transmission or its incidence in northern Cameroon, in the Senegal River valley, and in the Kou valley in Burkina Faso [6-9]. Most of these studies limit themselves in explaining aspects of relation between irrigated rice cultivation, transmission level, Plasmodium infection and malaria morbidity at the local level. However, to predict the consequences of rice cultivation development and to control its possible negative aspects, it is necessary to improve understanding of the interrelations between irrigated zones, environment and public health [10].

Thus, an interdisciplinary study of relationships between rice cultivation systems and malaria was conducted between 1997 and 1999 at regional level in three West African important settings: Sahel, savannah and forest. The Sahel study carried out in Mali showed that rice cultivation altered the transmission pattern from seasonal to perennial and reduced the incidence of malaria fevers in children aged less than 14 years [11]. In savannah in Côte d’Ivoire, irrigated rice cultivation did not modify the annual incidence of malarial attacks despite its seasonal influence on malaria risk [12].

The present study was carried out thirteen years ago in the forest region of western Côte d’Ivoire. Its objective was to compare malaria burden in three inland valley rice production systems in terms of prevalence and parasite density of infection, and also in terms of clinical malaria incidence. Furthermore the results of the present study may be put in perspective with the situation in Côte d’Ivoire after implementation of malaria control strategies.

## Methods

### Study zone

**Figure 1:**
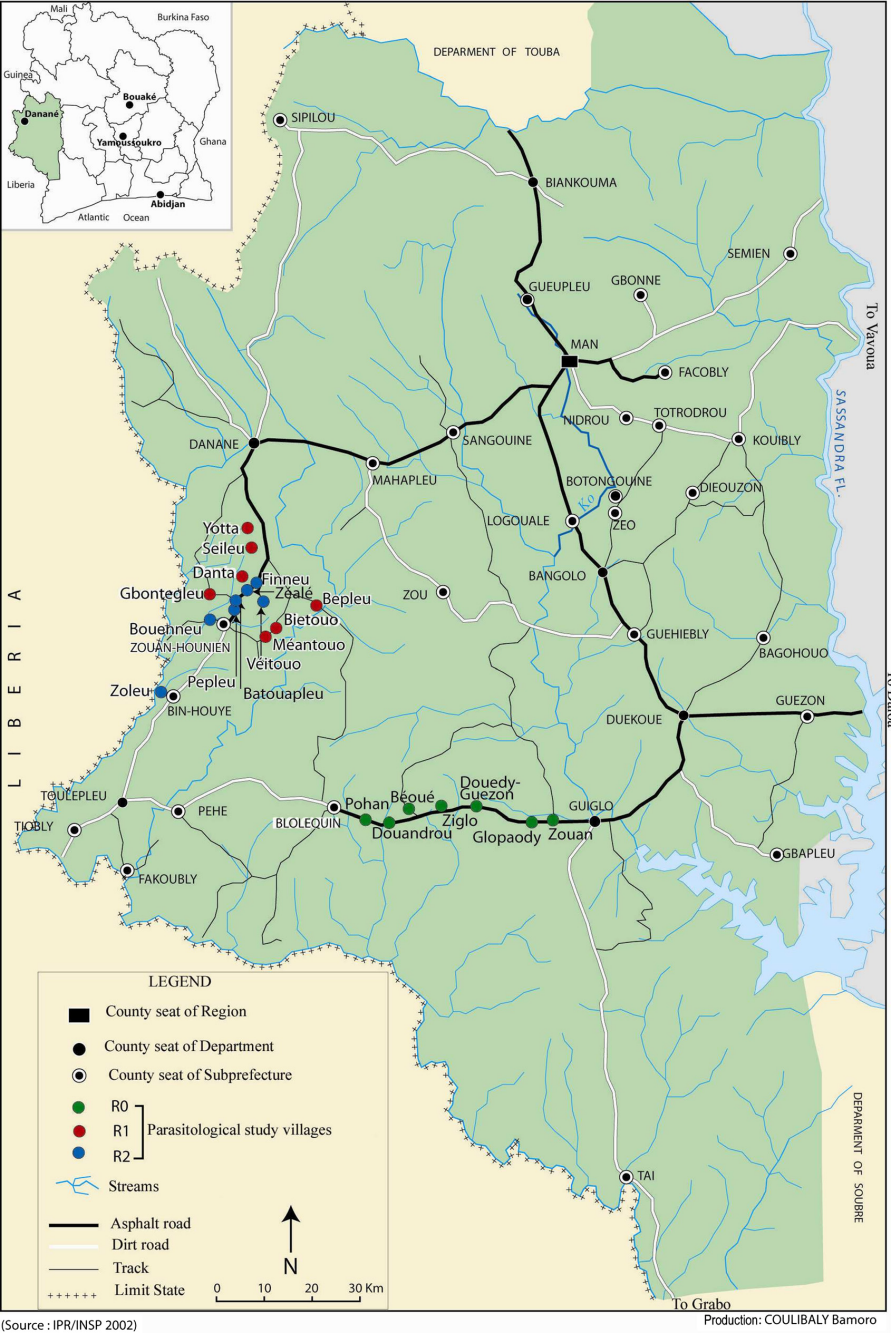
**Map of the study area showing the geographic position of the villages classified by rice farming systems (R0: no rice cultivation.** R1: single rice cropping and R2: double rice cropping).

A longitudinal study was carried out in the forest zone in the west of Côte d’Ivoire (Figure 1). The methodology for selecting the study villages was reported by Briët and colleagues [13]. All the villages were classified according to the farming systems in their surrounding inland valleys within a two kilometres radius: (R0) no (rice) cultivation; (R1) no water control, suitable for one cycle of rice cropping during the rainy season; (R2) partial or full water control which permits two cycles of rice cropping per year. The three categories of farming practice are referred to below as “agro-ecosystems”. Seven villages of each agro-ecosystem (R0, R1 and R2) were randomly selected among 97 villages pooled by farming system. All R1 and R2 villages were situated in the Department of Danané where all inland valleys were cultivated. The R0 villages where inland valleys were not at all farmed were situated in the Department of Guiglo close by. In the West of Côte d’Ivoire, most of the natural forest has been replaced by coffee and cocoa plantations. Moreover in the area south of Danané, numerous inland valleys were previously improved for rice cultivation. However water control was only partial in the inland valleys because of lack of maintenance. In this area, population belonged mostly to the Yacouba ethnic group. In the region west of Guiglo, population belonged to the Guéré ethnic group. Rainfall regime is long-monomodal with a maximum in September and a minimum in January [14]. Only the village of Zéalé has a centre of health.

### Sample size calculation

The same methodology of sampling as the one used in the savannah area was applied here [12]. The population required was estimated at 80,000 person-days to detect a relative risk below 0.5 by Poisson regression analysis with a power of 80%, a significance level of 5%, a maximum incidence of 0.64 malarial attacks per person-year [15] in the reference ecosystem and including 30% of participants lost to follow-up. This sample size was attained by selecting 285 persons in each village and monitoring them clinically for 40 days distributed over one year. People were selected from randomly sampled compounds. Children born during the study period were not included. Each family head and each person included in the study or their legal guardian gave informed consent. Ethical approval for the project was given by the Ivorian Ministry of Public Health and Hygiene. During the monitoring periods, patients of villages participating or not in the study were treated free of charge by the medical team.

### Parasitological and clinical measurements

Active case detection (ACD) for malaria episodes was done during eight periods of five consecutive days at six-weekly intervals in the year. During these periods the monitoring was made as described previously [12]. Briefly a nurse assisted by two health workers from the village who were recruited and trained for the purpose of the study visited every day all households covered by the study. A physician provided permanent supervision of the teams. The presence, absence and health condition of each included person were recorded daily by the nurse on a specially prepared form (one form per household). The nurse examined any detected sick person at home and registered clinical observations on an ad-hoc sheet. A thick blood film was taken systematically and patients were treated according to the clinical diagnosis made by the nurse. When malaria was suspected, the patient was treated with amodiaquine at the dose of 25 mg/kg body weight for three days in agreement with the guidelines of the National Malaria Control Programme [16]. Cross-sectional surveys (CSS) were held during each of the eight monitoring periods. A thick blood film was taken systematically from each asymptomatic participant (after checking the absence of fever) the second day of each period to make sure that he/she was free of illness during the days before and after the blood sampling.

### Laboratory procedures

Thick blood smears were stained with Giemsa in the field and examined using a microscope (ocular 10X, lens 100X) at Institut Pierre Richet (IPR) in Bouaké by six experienced technicians under the supervision of a parasitologist. *Plasmodium* species were identified and asexual forms of each species counted on 200 leucocytes. The parasite density was calculated by assuming an average of 8,000 leucocytes/μL of blood. A randomly selected 10% sample of the thick smears was double-read for quality control. The readings of the six technicians were compared using a set of blood smears.

### Data analysis

Demographic, clinical, parasitological and attendance data were double-entered independently in an Access database (ver.7 1995). Data were analysed using Stata V11 software. Only one blood sample per person per monitoring period was considered for the analysis. When a pathological condition was detected, it was the blood sample taken during the clinical episode that was considered for the analysis. When several blood samples from an asymptomatic period were available, one of them was randomly selected for the analysis. Parasitological data were analysed separately in terms of (1) prevalence of *Plasmodium falciparum*, *Plasmodium malariae* and *Plasmodium ovale* asexual blood forms, (2) density of *P. falciparum* asexual blood forms in parasite-positive thick smears, and (3) prevalence of *P. falciparum* gametocytes. A generalized estimating equation (GEE) approach was used for statistical analysis of repeated measures [17]. The GEE approach allows some departure from the hypothesis about the distribution of the dependent variable and gives robust estimates of regression coefficients taking into account the interdependence of observations made within the same person.

The prevalence of asymptomatic malaria infection and the prevalence of *P. falciparum* gametocyte presence were analyzed as a binomial response in logistic regression model. The parasite density was log transformed and analysed with a link function for a linear regression model. These two dependent variables were analysed according to demographic (age groups 0–1, 2–4, 5–9, 10–19, 20–39 and > =40 years), seasonal and agro-ecosystem variables.

The association between the parasite density and the occurrence of clinical episodes was tested using a random effect logistic regression model for each agro-ecosystem, taking clinical status (pathological episode *versus* asymptomatic state) as dependent variable, and parasite density, age and season as independent variables. In this type of model, a random intercept variable is allowed to vary with subjects and this random subject-specific intercept allows taking into account the interdependency of observations made on the same person. For each clinical episode, the probability that it was caused by malaria was estimated by the Attributable Fraction (AF) calculated from the odds ratios associated with the estimated parasite density in each logistic model [18]. The pathological episodes were defined by a high axillary temperature (≥37.5°C), a feeling of fever, sweat, shiver, headache, nausea or vomiting [19] or by a history of fever during the preceding 48 hours or for infants, anorexia or any pathological condition described by the mother [20].

For individuals the number of malarial attacks over a given period was estimated by the sum of probabilities that pathological episodes were due to malaria, depending on the parasite density. The malaria incidence density was calculated through the ratio of estimated number of malaria attacks and villagers’ person-days present during each monitoring period. The clinical malaria incidence in a standardized population was calculated, for each agro-ecosystem, by multiplying the averaged age specific incidence densities estimated in each agro-ecosystem with the number of subjects belonging to age groups of a virtual population of 1,000 persons with an age-distribution identical to the age-distribution of the populations of the three studied agro-ecosystems as a whole. Clinical malaria incidence densities observed in the different agro-ecosystems, age groups and seasons were compared using the likelihood ratio statistic in a Poisson regression model, with the estimated number of malarial attacks as dependent variable and the cumulative number of monitoring days as exposure variable.

## Results

### Population description

A total of 5,771 persons were selected in the three agro-ecosystems, 1,915, 1,971 and 1,885 in the villages R0, R1 and R2, respectively. The study was carried out between May 98 and March 99. The average of age of people was 28 (27–29) years in R0 and 22 (21–23) years in R1 and R2. The female/male ratio was 1.20 in R0, 1.02 in R1 and 1.03 in R2 (Table 1). These people were clinically and parasitologically monitored during 46,168 person-weeks of which 34% were lost for different reasons: 22% not found because inhabitants were working in camps far from the village during the passage of the medical team and 10% of refusals because people feared that thick blood films of healthy persons could be used for magic. One hundred ten persons (1.2%) died. There were 0.8% of missing observations mainly because of unknown clinical status (Table 2). The villages Vetouo, Finneu and Zoleu classified as R2 (based on previous years) had little or no off-season rice cultivation during the study because water control is only partial in these inland valleys, and as a result, the agricultural calendar is strongly dependent on rainfall [13].

**Table 1:** Distribution of population sample by age group according to villages and rice farming systems

**Agro-ecosystem**	**Villages**	**0-1 year**	**2-4 years**	**5-9 years**	**10-19 years**	**20-39 years**	**> = 40 years**	**Total**	**Geographic position**	
									**Lat. North**	**Long. West**
**R0**	Pohan	8	29	40	60	57	80	274	6°32’59”	7°56’22”
	Douandrou	5	28	48	43	65	7	259	6°32’16”	7°54’39”
	Ziglo	16	32	38	41	73	84	284	6°33’29”	7°48’26”
	Zouan	12	30	42	58	62	76	280	6°32’34”	7°35’33”
	Douedy-Guezon	7	20	47	62	57	80	273	6°33’50”	7°45’43”
	Beoue	10	34	46	49	68	72	279	6°32’43”	7°38’58”
	Glopaoudy	16	28	31	44	69	78	266	6°32’43”	7°38’07”
	**Total**	**74**	**201**	**292**	**357**	**451**	**540**	**1,915**		
**R1**	Danta	7	39	56	74	96	61	333	7°01’16”	8°10’11”
	Yotta	12	22	47	63	71	58	273	7°05’13”	8°10’48”
	Bepleu	12	23	63	65	70	57	290	6°56’48”	8°01’47”
	Seileu	17	21	55	47	89	42	271	7°02’51”	8°10’37”
	Bietouo	10	45	51	55	71	45	277	6°54’20”	8°07’25”
	Meantouo	14	31	47	72	57	39	260	6°55’09”	8°06’57”
	Gbontegleu	15	34	42	45	85	46	267	6°58’15”	8°13’51”
	**Total**	**87**	**215**	**361**	**421**	**539**	**348**	**1,971**		
**R2**	Zealé	6	28	38	56	88	58	274	6°59’07”	8°09’28”
	Zoleu	15	36	55	45	74	44	269	7°03’30	8°10’03”
	Finneu	11	30	53	50	72	54	270	7°00’57”	8°08’43”
	Pepleu	14	36	53	63	65	41	272	6°56’27”	8°11’36”
	Vetouo	9	25	61	54	66	60	275	6°57’31”	8°06’58”
	Batouapleu	15	26	44	75	46	38	244	6°57’40”	8°11’32”
	Boueneu	17	19	53	71	76	45	281	6°55’20	8°13’57”
	**Total**	**87**	**200**	**357**	**414**	**487**	**340**	**1,885**		
	**Total**	**248**	**616**	**1,010**	**1,192**	**1,477**	**1,228**	**5,771**		

*R0* no rice cultivation, *R1* single rice cropping and *R2* double rice cropping.

**Table 2:** Participation in monitoring

	**R0**	**R1**	**R2**	**Total**
N selected persons	1,915	1,971	1,885	5,771
N expected observations person-week*	15,320	15,768	15,080	46,168
Refusal	1,876	1,134	1,610	4,620 (10%)
Dead	262	131	161	554 (1.2%)
Lost of view	4,541	2,903	2,710	10,154 (22%)
Missing information	118	140	109	367 (0.8%)
N effective observations person-week	8,523	11,470	10,490	30,483
Asymptomatic subjects	8,189	11,020	10,121	29,330
Sick patients	334	450	369	1,153

* N people x 8 surveys.

### Parasitological indexes of asymptomatic subjects observed during CSS

*Plasmodium falciparum* was largely predominant on *P. malariae* and *P. ovale* in the three agro-ecosystems. The mean annual prevalence of *P. falciparum* asymptomatic infections was 48.1% (95% CI: 44.8-51.4) in R0, 44.9% (95% CI: 41.7-48.2) in R1 and 47.1% (95% CI: 45.6-48.7) in R2 (Table 3). In the multivariate GEE logistic regression model, age of asymptomatic subjects, but not the season, were significantly associated with the prevalence of infection (Table 4). Everywhere, more than 75% of children aged 0 to 4 years were parasite-positive. From five years, the prevalence of infection quickly decreased with age. The subjects of 5–9 years, of 10–19 years and of 20 years and more were 2, 4 and 10 times less infected than the infants, respectively. The prevalence of infection was slightly lower in the R1 farming system than in the R0 farming system (P = 0.025) and did not differ significantly between the R0 and the R2 farming systems.

**Table 3:** Prevalence of plasmodial infection in asymptomatic subjects according to farming systems (R0: no rice cultivation; R1: single rice cropping; R2: double rice cropping) and age groups

			***Plasmodium falciparum *infection**					***Plasmodium malariae *infection**		***Plasmodium ovale *infection**	
			**Asexual blood stages**			**Gametocytes**		**Asexual blood stages**		**Asexual blood stages**	
**Agro-ecosystem**	**Age groups (years)**	**N**	**Positive thick blood smear**	**Prevalence rate (95% CI)**	**Geometric mean density (95% CI)**	**Positive thick blood smear**	**Prevalence rate (95% CI)**	**Positive thick blood smear**	**Prevalence rate (95% CI)**	**Positive thick blood smear**	**Prevalence rate (95% CI)**
**R0**	**0–1**	291	219	75.3 (70.4–80.1)	903 (702–1162)	33	11.3 (7.8–14.9)	27	9.3 (5.9–12.6)	3	1.0 (0.1–2.2)
	**2–4**	830	637	76.7 (71.8–81.7)	578 (513–651)	46	5.5 (3.6–7.5)	72	8.7 (6.8–10.6)	15	1.8 (0.9–2.7)
	**5–9**	1,263	830	65.7(61.3670.1)	237 (216–259)	29	2.3 (1.5–3.1)	45	3.6 (2.5–4.6)	5	0.4 (0.0–0.7)
	**10–19**	1,482	748	50.5 (45.5–55.4)	134 (123–147)	19	1.3 (0.7–1.9)	27	1.8 (1.1–2.5)	3	0.2 (0.0–0.4)
	**20–39**	1,884	700	37.2 (32.6–41.7)	93 (82–105)	26	1.4 (0.9–1.9)	10	0.5 (0.2–0.9)	2	0.1 (0.0–0.3)
	**> = 40**	2,439	806	33.0 (29.6–36.5)	81 (69–94)	18	0.7 (0.2–1.3)	10	0.4 (0.2–0.7)	0	0.0 (0.0–0.0)
	**Total**	8,189	3,940	48.1 (44.8–51.4)	180 (165–195)	171	2.1 (1.8–2.4)	191	2.3 (2.0–2.7)	28	0.3 (0.2–0.5)
**R1**	**0–1**	426	325	76.3 (70.3–82.3)	843 (583–1220)	33	7.7 (3.3–12.2)	36	8.5 (5.8–11.1)	8	1.9 (0.6–3.2)
	**2–4**	1,207	926	76.7 (74.0–79.4)	669 (551–812)	79	6.5 (4.7–8.4)	95	7.9 (6.4–9.4)	11	0.9 (0.4–1.4)
	**5–9**	1,997	1,259	63.0 (59.3–66.8)	225 (193–262)	76	3.8 (2.2–5.4)	46	2.3 (1.6–3.0)	4	0.2 (0.0–0.4)
	**10–19**	2,194	932	42.5 (38.0–47.0)	120 (107–135)	32	1.5 (0.7–2.3)	12	0.5 (0.2–0.9)	6	0.3 (0.1–0.5)
	**20–39**	3,107	965	31.1 (27.4–34.7)	81 (70–94)	31	1.0 (0.5–1.5)	12	0.4 (0.2–0.6)	1	0.0 (0.0–0.1)
	**> = 40**	2,089	545	26.1 (21.9–30.3)	71 (59–86)	20	1.0 (0.6–1.4)	15	0.7 (0.4–1.1)	0	0.0 (0.0–0.0)
	**Total**	11,020	4,952	44.9 (41.7–48.2)	193 (194–219)	271	2.5 (2.2–2.7)	216	2.0 (1.7–2.2)	30	0.3 (0.2–0.4)
**R2**	**0–1**	448	346	77.2 (73.4–81.1)	943 (765–1163)	40	8.9 (6.5–11.3)	29	6.5 (4.2–8.8)	5	1.1 (0.1–2.1)
	**2–4**	1,031	812	78.8 (76.5–81.1)	614 (540–698)	64	6.1 (4.9–7.3)	78	7.6 (6.0–9.2)	20	1.9 (1.1–2.8)
	**5–9**	2,008	1,256	62.5 (60.4–64.7)	243 (225–263)	56	2.8 (2.1–3.5)	35	1.7 (1.2–2.3)	5	0.2 (0.0–0.5)
	**10–19**	2,097	982	46.8 (44.5–49.1)	137 (126–149)	36	1.7 (1.2–2.3)	16	0.8 (0.4–1.1)	5	0.2 (0.0–0.4)
	**20–39**	2,617	821	31.4 (29.7–33.7)	91 (82–100)	16	0.6 (0.3–0.9)	9	0.3 (0.1–0.6)	3	0.1 (0.0–0.2)
	**> = 40**	1,920	555	28.9 (26.7–31.1)	77 (71–84)	10	0.5 (0.3–0.8)	10	0.5 (0.2–0.8)	1	0.1 (0.0–0.2)
	**Total**	10,121	4,772	47.1 (45.6–48.7)	206 (194–219)	222	2.2 (1.9–2.5)	177	1.7 (1.5–2.0)	39	0.4 (0.3–0.5)

**Table 4:** **Multivariate analysis of the prevalence rates of *Plasmodium falciparum *asymptomatic infection determined by cross-sectional survey**

	**N**	**Positive thick blood smear**	**Prevalence rate (95% CI)**	**Unadjusted odds-ratio (95% CI)**	**p-values**	**Adjusted odds-ratio (95% CI)**	**p-values**
**Agro-ecosystem**					0.358		0.081
**R0**	8,189	3,940	48.1 (44.8–51.4)	1.00		1.00	
**R1**	11,020	4,952	44.9 (41.7–48.2)	0.88 (0.73–1.06)	0.166	0.79 (0.64–0.97)	0.025
**R2**	10,121	4,772	47.1 (45.6–48.7)	0.96 (0.83–1.11)	0.583	0.85 (0.73–1.00)	0.053
**Age group (years)**					<0.0001		<0.0001
**0–1**	1,165	890	76.4 (73.5–79.3)	1.00		1	
**2–4**	3,068	2,375	77.4 (75.5–79.3)	1.06 (0.88–1.27)	0.525	1.06 (0.88–1.27)	0.518
**5–9**	5,268	3,345	63.5 (61.6–65.4)	0.54 (0.45–0.65)	<0.0001	0.54 (0.45–0.65)	<0.0001
**10–19**	5,773	2,662	46.1 (43.7–48.6)	0.26 (0.22–0.32)	<0.0001	0.26 (0.22–0.32)	<0.0001
**20–39**	7,608	2,486	32.7 (30.6–34.7)	0.15 (0.12–0.18)	<0.0001	0.15 (0.12–0.18)	<0.0001
**> = 40**	6,448	1,906	29.6 (27.5–31.6)	0.13 (0.11–0.16)	<0.0001	0.13 (0.10–0.16)	<0.0001
**Season**					0.741		0.705
**Dry**	10,537	4,883	46.3 (44.9–48.4)	1.00		1.00	
**Wet**	18,793	8,781	46.7 (44.7–48.8)	1.01 (0.92–1.11)	0.741	1.02 (0.92–1.13)	0.705

The geometric mean parasite density in positive asymptomatic persons varied in the three agro-ecosystems between 180 and 206 *P. falciparum* asexual forms per μL of blood. According to multivariate GEE linear regression model, parasite density was associated with season and with age, but not with farming system (Table 5). Parasite density was higher in the rainy season than in the dry season (208 (95% CI: 190–227) *versus* 171 (95% CI: 159–184)) asexual forms per μL of blood and significantly decreased with age.

**Table 5:** Multivariate analysis of parasite densities among positive asymptomatic subjects observed by cross-sectional survey

	**N**	**Positive thick blood smear**	**Geometric mean (95% CI)**	**Unadjusted multiplicative coefficient (95% CI)**	**p-values**	**Adjusted multiplicative coefficient (95% CI)**	**p-values**
**Agro ecosystem**					0.0478		0.401
**R0**	8,189	3,940	180 (165–195)	1.00		1.00	
**R1**	11,020	4,952	193 (171–217)	1.07 (0.93–1.24)	0.325	0.95 (0.84–1.08)	0.434
**R2**	10,121	4,772	206 (194–219)	1.15 (1.03–1.27)	0.013	1.02 (0.93–1.11)	0.694
**Age groups (years)**					< 0.0001		<0.0001
**0–1**	1,165	890	895 (755–1059)	1.00		1.00	
**2–4**	3,068	2,375	624 (569–684)	0.70 (0.56–0.87)	0.003	0.70 (0.56–0.87)	0.004
**5–9**	5,268	3,345	234 (218–251)	0.26 (0.22–0.32)	< 0.0001	0.26 (0.22–0.32)	<0.0001
**10–19**	5,773	2,662	130 (122–138)	0.15 (0.12–0.17)	< 0.0001	0.14 (0.12–0.17)	<0.0001
**20–39**	7,608	2,486	87 (81–94)	0.10 (0.08–0.12)	< 0.0001	0.10 (0.08–0.12)	<0.0001
**> = 40**	6,448	1,906	77 (70–84)	0.09 (0.07–0.10)	< 0.0001	0.09 (0.07–0.10)	<0.0001
**Season**					0.0057		0.0085
**Dry**	10,537	4,883	171 (159–184)	1.00		1.00	
**Wet**	18,793	8,781	208 (190–227)	1.22 (1.07–1.39)	0.006	1.21 (1.05–1.38)	0.009

The mean annual gametocyte rates were similar between the 3 farming systems, 2.1% (95% CI: 1.8-2.4) in R0, 2.5% (95% CI: 2.2-2.7) in R1 and 2.2% (95% CI: 1.9-2.5) in R2, respectively. The highest rates were observed in the 0–4 old children (P < 0.0001). Prevalence of *P. falciparum* gametocytes was higher in the rainy season than in the dry season (OR: 1.45; 95% CI: 1.16-1.81; P = 0,003).

The plasmodial formula on the 13,746 *Plasmodium*-positive thick blood films of asymptomatic subjects was 0.994, 0.042 and 0.007 for respectively *P. falciparum*, *P. malariae* and *P. ovale*. According to multivariate GEE linear regression model, the prevalence of *P. malariae* infections was similar in R0 (2.3%) and R1 (2.0%) (OR: 0.76 (95% CI: 0.52-1.11); P = 0,151), but slightly lower in R2 (1.7%) than in R0 (OR: 0.67 (95% CI: 0.48-0.93); P = 0.019). Children 0–4 years old were the most frequently infected (P < 0.0001). According to season prevalence of *P. malariae* species was higher in dry season (2.5%) than in wet season (1.7%) (OR: 0.66 (95% CI: 0.49-0.87); P = 0.006). The *P. ovale* asymptomatic infection rates were similar in the three agro-ecosystems (P = 0.564) and seasons (P = 0.654). Children 0–4 years old were the most frequently infected (P < 0.0001).

### Clinical malaria incidence observed by ACD method

**Figure 2:**
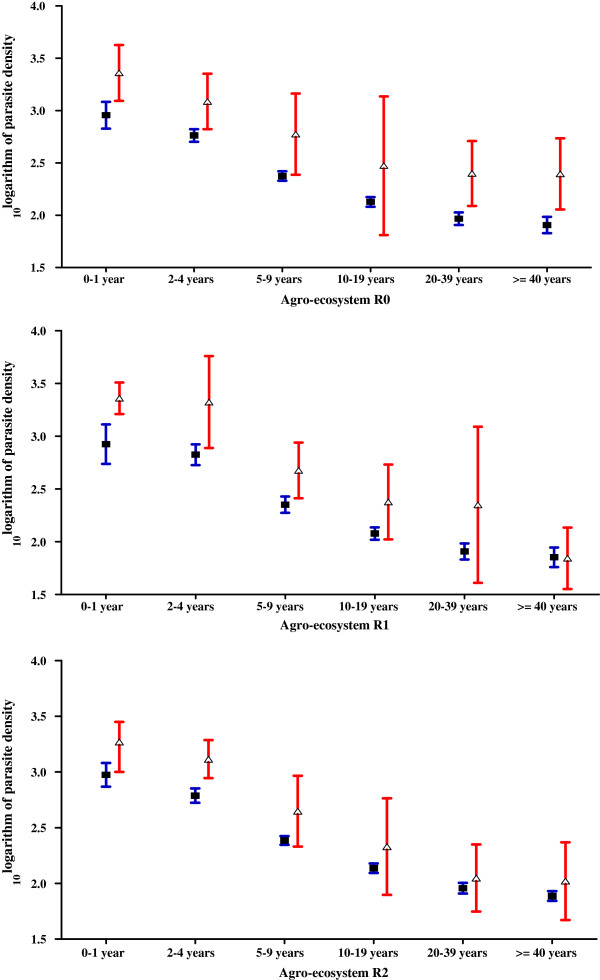
Annual means parasite density (95% CI) in parasite-positive asymptomatic subjects (observed during cross-section surveys) and patients (found by active detection method) according to age groups in the three farming systems.

During the study 1,153 pathological episodes were detected in the three agro-ecosystems. A total of 756 episodes were parasite positive: 695 individuals with *P. falciparum* alone, three with *P. malariae* alone and 58 individuals with mixed infection (48 *P. falciparum*, *P. malariae* positive infections, seven *P. falciparum/P. ovale* positive infections, three *P. falciparum*/*P. malariae/P. ovale* positive infections). The *P. malariae* infections showed densities of 100 and 40 parasites/μL. Among 47 out of the 58 mixed infections *P. falciparum* was more frequent than the other plasmodial species. Whatever the age group sick patients had higher geometric mean parasite densities than healthy people (Figure 2).

**Figure 3:**
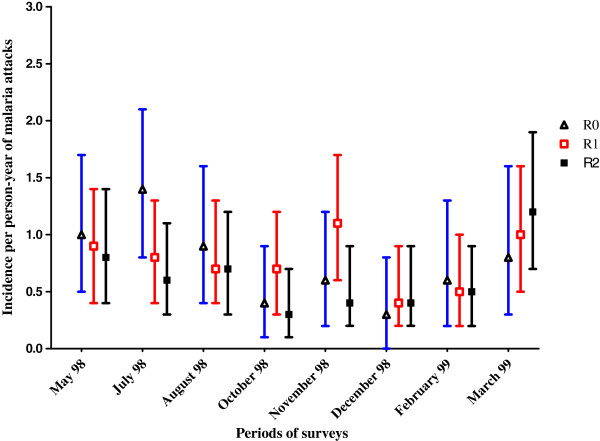
Clinical incidence rates (95% CI) in the three farming systems according to the surveys.

Among the 1,153 pathological episodes, there were 234 episodes attributable to *P. falciparum* malaria (Table 6). The mean annual incidence rate was 0.7 (95% CI: 0.5-0.9) in R0, 0.7 (95% CI: 0.6-0.9) in R1 and 0.6 (95% CI: 0.5-0.7) clinical malaria episode per person (Table 6). According to the Poisson regression model clinical incidence rates significantly varied with age and season but not with farming system (Table 7). The burden of malaria was the highest among children under two years of age, with at least 4 attacks by person-year. Then malaria incidence decreased by half in the two to four years age group. From the age of five years, the incidence was lower than one attack per person-year. The incidence of clinical malaria was significantly higher in the rainy season than in the dry season. The clinical incidence rates in all three farming systems varied nearly in the same way as the seasons went (Figure 3).

**Table 6:** Incidence density of pathological episodes and malaria attacks according to age and agro-ecosystem

	**Observation**			**Pathological episodes**		**Malaria episodes**			**Malaria fever in the standard population of 1000 persons**	
	**Age groups (years)**	**N**	**Person-day**	**N**	**Incidence per person-year (95% CI)**	**N**	**Attributable fraction (95% CI)**	**Incidence per person-year**	**Population (%)**	**Incidence per year (95% CI)**
**R0**	**0–1**	390	1,532	66	15.7 (8.8–25.4)	55	22.5 (14.2–33.9)	5.4 (3.4–8.1)	7.4%	397 (358–438)
	**2–4**	1,008	3,909	89	8.3 (3.5–15.8)	73	24.6 (15.8–36.3)	2.3 (1.5–3.4)	9.8%	225 (197–256)
	**5–9**	1,443	5,243	43	3.0 (0.4–8.0)	32	9.1 (4.1–17.1)	0.6 (0.3–1.2)	17.5%	111 (91–133)
	**10–19**	1,701	5,919	21	1.3 (0.0–5.6)	7	1.0 (0.0–5.6)	0.1 (0.0–0.3)	21.4%	13 (07–22)
	**20–39**	2,180	7,901	59	2.7 (0.4–8.0)	31	4.7 (1.4–11.0)	0.2 (0.1–0.5)	19.9%	43 (31–58)
	**> = 40**	2,849	10,469	56	2.0 (0.1–6.4)	27	4.8 (1.4–11.0)	0.2 (0.0–0.4)	24.0%	40 (29–54)
	**Total**	9,571	34,973	334	3.5 (0.6–8.8)	225	66.7 (51.5–84.5)	0.7 (0.5–0.9)	100.0%	696 (645–750)
**R1**										
**0–1**		572	2,301	123	19.5 (11.8–30.3)	106	43.0 (30.7–57.3)	6.8 (4.9–9.2)	7.4%	505 (461–551)
**2–4**		1,371	5,534	95	6.3 (2.2–13.1)	80	30.3 (20.2–42.8)	2.0 (1.3–2.8)	9.8%	196 (169–225)
**5–9**		2,245	8,558	74	3.2 (0.6–8.8)	39	10.9 (5.1–19.0)	0.5 (0.2–0.8)	17.5%	81 (64–101)
**10–19**		2,484	9,080	45	1.8 (0.1–6.4)	22	4.9 (1.4–11.0)	0.2 (0.1–0.4)	21.4%	42 (30–57)
**20–39**		3,468	12,713	67	1.9 (0.1–6.4)	23	3.6 (0.8–9.5)	0.1 (0.0–0.3)	19.9%	21 (13–31)
**> = 40**		2,320	8,816	46	1.9 (0.1–6.4)	14	0.7 (0.0–4.7)	0.0 (0.0–0.2)	24.0%	07 (03–14)
**Total**		12,460	47,002	450	3.6 (0.6–8.8)	284	93.4 (75.1–113.9)	0.7 (0.6–0.9)	100.0%	725 (673–780)
**R2**										
**0–1**		566	2,441	92	13.8 (7.3–22.9)	80	31.1 (21.1–44.0)	4.7 (3.1–6.6)	7.4%	344 (309–382)
**2–4**		1,210	5,143	97	6.9 (2.5–13.7)	82	25.9 (16.6–37.5)	1.8 (1.2–2.7)	9.8%	180 (155–208)
**5–9**		2,260	8,999	58	2.4 (0.2–7.2)	33	11.0 (5.5–19.7)	0.4 (0.2–0.8)	17.5%	78 (62–97)
**10–19**		2,398	9,110	38	1.5 (0.1–6.4)	16	2.6 (0.4–8.0)	0.1 (0.0–0.3)	21.4%	22 (14–33)
**20–39**		3,000	11,561	52	1.6 (0.1–6.4)	21	2.7 (0.4–8.0)	0.1 (0.0–0.3)	19.9%	17 (10–27)
**> = 40**		2,213	8,693	32	1.3 (0.0–5.6)	15	0.9 (0.0–4.7)	0.0 (0.0–0.2)	24.0%	09 (04–17)
**Total**		11,647	45,947	369	2.9 (0.4–8.0)	247	74.2 (58.1–92.9)	0.6 (0.5–0.7)	100.0%	589 (542–639)

**Table 7:** Poisson regression analysis of malaria episodes observed by active case detection

	**N**	**Person day**	**Fraction attributable**	**Relative risk (95% CI)**	**p-values**
**Farming system**					0.1524
**R0**	9,571	34,973	66.7	1.00	
**R1**	12,460	47,002	93.4	0.93 (0.68–1.26)	0.633
**R2**	11,647	45,947	74.2	0.74 (0.54–1.02)	0.066
**Age groups (years)**					< 0.0001
**0–1**	1,528	6,274	96.6	1.00	
**2–4**	3,589	14,586	80.7	0.38 (0.28–0.50)	< 0.0001
**5–9**	5,948	22,800	31.1	0.09 (0.06–0.14)	< 0.0001
**10–19**	6,583	24,109	8.6	0.02 (0.01–0.04)	< 0.0001
**20–39**	8,648	32,175	11.0	0.02 (0.02–0.04)	< 0.0001
**> = 40**	7,382	27,978	6.4	0.03 (0.02–0.05)	< 0.0001
**Season**					0.0078
**Dry**	12,305	4,7038	66.8	1.00	
**Wet**	21,373	80,884	167.6	1.46 (1.10–1.92)	0.008

## Discussion

This prospective longitudinal study carried out in western Côte d’Ivoire allowed to measure in the forest area the impact of inland valley rice cultivation on malaria infection and disease.

### Epidemiology

A level of holoendemicity of malaria was observed in the three agro-ecosystems, R0 where no rice was cultivated, R1 with single rice cropping and R2 with double rice cropping, respectively. In all three agro ecosystems more than 75% of children less than 12 months old were infected by *P. falciparum*[21]. During nearly the same period of the present study six entomological surveys were performed in two villages of each agro ecosystem [22]. A high perennial transmission was observed with annual entomological inoculation rates (EIR) of 146, 333 and 520 infective bites per human in R0, R1 and R2, respectively. Similar levels of transmission were reported in the same area where considerable ecological changes linked to the deterioration of the forest environment occurred over the past 30 years [23,24]. In the three agro-ecosystems *An. gambiae* s.l. and *Anopheles funestus* s.l*.* were the main vectors, with a predominance of *An. gambiae*[22]. The *An*. *gambiae* population density was strongly correlated with the surface water availability in the rice-cultivated inland valleys, but weakly correlated with the surface water availability in the uncultivated inland valleys [13]. Moreover the *An. funestus* population density and its rate of infected bites were found to increase during the dry season but only in the rice farming systems and especially in R2 [22]. Thus the inland valley rice cultivation in the forest area appeared as a risk factor for malaria transmission. Nevertheless no effect was observed on the malaria infection and disease. The asymptomatic infections showed similar annual mean prevalence rates and annual mean parasite densities in the three farming systems. The annual mean rates of clinical incidence were also similar with 0.7 malaria attacks per person in R0 and R1 and 0.6 in R2. This paradox could be explained: (i) according to the model of Smith and colleagues [25], at a high level of transmission the parasite prevalence does not vary much anymore, even if the EIR increases further, (ii) people of the R0 agro ecosystem might be infected outside their villages because they lived more in camps to work in the coffee and cocoa plantations than people of other both rice farming systems. A high rate of absence of inhabitants was observed during the surveys of the medical team, especially in the R0 villages (18% in R1 and R2 and 30% in R0).

Other species of Plasmodium, such as *P. malariae* and *P. ovale,* were also found in the three agro-ecosystems, but with low rates of prevalence, 2% and 0.3%, respectively.

Irrespective of the agro-ecosystem, inhabitants experienced on average three pathological episodes a year of which 20% were falciparum clinical malaria attacks. Thus, malaria disease represented 600–700 episodes a year in the standard population of 1,000 persons (all age groups). To quantify malaria morbidity, the method of estimation of the fever fraction attributable to malaria was used [18]. In the forest area where malaria transmission was around one infected bite per person per night, the mean parasite densities strongly differed between sick and asymptomatic persons. Even if the distribution of parasite infection in asymptomatic subjects includes a number of highly infected individuals, the attributable fraction can be estimated [20,26].

In the three farming systems, clinical malaria decreased strongly and quickly with age. Infants were more affected than children and from the age of five years, people experienced less than one episode a year. All the parasitological and clinical findings indicate that the naturally acquired immunity to falciparum malaria starts at a very early age and the majority of infected adults and older children rarely experience overt disease [27]. Indeed most people are almost continuously infected by *P. falciparum* because the transmission is intense throughout the year, with a peak during the rainy season [23]. The parasitological and clinical indices were higher in the rainy season than in the dry season, except for the prevalence rate of asymptomatic infections for which the seasonal difference was low and not significant. It is the consequence of a seasonal increase of the transmission that is perennial.

### Parasitological and clinical comparison with other sites

In the forest area the inland valley rice cultivation did not influence the rates of malaria infection and disease although it appeared as a risk for malaria transmission. An economic study carried out on 750 households (21 villages) in the three agro-ecosystems also showed that malaria had no effect on coffee and cocoa productions and that lowland irrigated rice cultivation was not a risk factor for malaria infection [28].

People living in the R0 agro ecosystem where no inland valley was cultivated spent probably more time in the camp for working in the coffee and cocoa plantations than in the other agro systems. This situation could explain the high rate of absence of inhabitants during the surveys of the medical team (18% in R1 and R2 and 30% in R0). Despite this potential behavioural difference and the differences in ethnic group of the populations, there was no significant difference in malaria burden between the three agro-ecosystems.

Two other studies carried out within the framework of the Health Research Consortium hosted by the West African Rice Development Association (WARDA) (currently known as the Africa Rice Center), following a North–south transect in West Africa showed quite different results. In Côte d’Ivoire in the savannah zone where the same methodology as in the forest zone was used, malaria was hyper-endemic. The mean annual incidence rate reached 0.9 (95% IC: 0.8-1.0) clinical malaria episodes per person in R0, 0.6 (95% CI: 0.5-0.7) in R1 and 0.8 (95% CI: 0.7-1.0) in R2 [12]. The development of areas for rice cultivation did not modify the annual incidence of malarial attacks despite the seasonal influence on malaria risk [12]. However the lower malaria morbidity rate in R1 was more attributable to specific economic behaviour than to rice production systems. According to a study on the property accumulation in rice production systems [28], the agricultural households in R0 were less rich than those in R1 because of the poor results obtained from cotton cultivation rather than to the lack of rice cultivation. The agricultural households in R2 were also less rich than those in R1 which were cotton producers and invested the most in equipment. Moreover the fields in R2 were much smaller than those in R1, the reason being that double rice cropping was exhausting [29]. Indeed this farming system asked working during the whole year whereas households in R1 were cultivating during only six months. On the other hand the socio-economic transformations and the gender repositioning induced, or facilitated, by the intensification of inland valley irrigated rice cultivation led to a reduction of the capacity of women to manage disease episodes, contributing therefore to increase malaria incidence among farming populations [29,30]. More specifically, Audibert *et al.*[31,32] showed that malaria had a negative effect on productivity and income and constituted a barrier for property accumulation in savannah.

The study carried out in Mali in the semi-arid sub-Saharan environment showed that irrigated rice cultivation altered the malaria transmission pattern from seasonal to perennial and reduced annual morbidity incidence more than two-fold, compared to cultivation without irrigation [11].

## Conclusion

This study, together with the other studies conducted in the framework of the Health Research Consortium in the forest, the savannah and the Sahel were performed on a large scale with a similar methodology in the context of farming systems rather than in single villages. The findings in these ecologically different zones (forest, savannah and Sahel) clearly confirm the conclusion that Carnevale and colleagues [3] and Ijumba and Lindsay [1] drew from studies carried out in different sites such as Burundi [4], Burkina Faso [9], Senegal [7], The Gambia [33]: in a stable malaria zone irrigated rice cultivation does not significantly affect malaria pressure, contrary to what may occur in an unstable malaria zone.

The study also provides an important basic data set before Côte d’Ivoire implemented the Roll Back Malaria strategies. It should permit to put the results of the current malaria control programme in Côte d’Ivoire into perspective.

## Competing interests

The authors declare that they have no competing interests.

## References

[B1] Ijumba JN Lindsay SW Impact of irrigation on malaria in Africa: paddies paradox Med Vet Entomol 2001 15 1 11 10.1046/j.1365-2915.2001.00279.x 11297093

[B2] Oomen JMV de Wolf J Jobin WR Heath and irrigation. Incorporation of disease-control measures in irrigation. A multi-faceted task in design. Construction. Operation 1994 Wageningen, The Netherlands: International Institute for Land Reclamation and Improvement (ILRI)

[B3] Carnevale P Guillet P Robert V Fontenille D Doannio J Coosemans M Mouchet J Diversity of malaria in rice growing areas of the Afrotropical region Parassitologia 1999 41 273 276 10697868

[B4] Coosemans MH Comparison of malarial endemicity in a rice-growing area and a cotton-growing area of the Rusizi Plain (Burundi) Ann Soc Belg Med Trop 1985 65 187 200 4073976

[B5] Laventure S Mouchet J Blanchy S Marrama L Rabarison P Andrianaivolambo L Rajaonarivelo E Rakotoarivony I Roux J Le Riz: source de vie et de mort sur les plateaux de Madagascar (in French) Cahiers Santé 1996 6 79 86 8705134

[B6] Audibert M Josseran R Josse R Adjidji A Irrigation. Schistosomiasis and malaria in the Logone Valley, Cameroon Am J Trop Med Hyg 1990 42 550 560 2115305 10.4269/ajtmh.1990.42.550

[B7] Faye O Fontenille D Hervé JP Diack PA Diallo S Mouchet J Malaria in the Saharan region of Senegal. 2. Parasitological indices Ann Soc Belg Med Trop 1993 73 31 36 8323406

[B8] Faye O Fontenille D Gaye O Sy N Molez JF Konaté L Hebrard G Hervé JP Trouillet J Diallo S Paludisme et riziculture dans le delta du fleuve Sénégal (Sénégal) Ann Soc Belg Med Trop 1995 75 179 189 8849295

[B9] Boudin C Robert V Carnevale P Ambroise-Thomas P Epidemiology of *Plasmodium falciparum* in a rice field and a savannah area in Burkina Faso. Comparative study on the acquired immunoprotection in native populations Acta Trop 1992 51 103 111 10.1016/0001-706X(92)90052-Y 1354928

[B10] Gioda A Identical causes but various effects: irrigation, health and development Sécheresse 1992 3 227 23423858558

[B11] Sissoko MS Dicko A Briët OJ Sissoko M Sagara I Keita HD Sogoba M Rogier C Touré YT Doumbo OK Malaria incidence in relation to rice cultivation in the irrigated Sahel of Mali Acta Trop 2004 89 161 170 10.1016/j.actatropica.2003.10.015 14732238

[B12] Henry MC Rogier C Nzeyimana I Assi SB Dossou-Yovo J Audibert M Mathonnat J Keundjian A Akodo E Teuscher T Carnevale P Inland valley rice production systems and malaria infection and disease in the savannah of Côte d’Ivoire Trop Med Int Health 2003 8 449 458 10.1046/j.1365-3156.2003.01053.x 12753641

[B13] Briët OJT Dossou-Yovo J Akodo E van de Giesen N Teuscher TM The relationship between *Anopheles gambiae* density and rice cultivation in the savannah zone and forest zone of Côte d’Ivoire Trop Med Int Health 2003 8 439 448 10.1046/j.1365-3156.2003.01054.x 12753640

[B14] Goula BTA Srohourou B Brida AB Kanga BI Goroza G Zoning of rainfall in Côte d’Ivoire Int J Eng Sci Tech 2010 2 6004 6015

[B15] Trape JF Rogier C Konaté L Diagne N Bouganali H Canque B Legros F Badji A Ndiaye G Ndiaye P Brahimi K Druilhe P Da Silva LP The Dielmo project: a longitudinal study of natural malaria infection and the mechanisms of protective immunity in a community living in a holoendemic area of Senegal Am J Trop Med Hyg 1994 51 123 137 8074247 10.4269/ajtmh.1994.51.123

[B16] Henry MC Niangué J Koné M Quel médicament pour traiter le paludisme simple quand la chloroquine devient inefficace dans l’Ouest de la Côte d’Ivoire? Méd Trop 2002 62 55 57 12038180

[B17] Zeger SL Liang K Longitudinal data analysis for discrete and continuous outcomes Biometrics 1986 42 212 130 3719049

[B18] Schellenberg JR Smith T Alonso PL Hayes RJ What is clinical malaria? Finding case definition for field research in highly endemic areas Parasitol Today 1994 10 439 442 10.1016/0169-4758(94)90179-1 15275531

[B19] Rogier C Ly AB Tall A Cisse B Trape JF *Plasmodium falciparum* clinical malaria in Dielmo, a holoendemic area in Senegal: no influence of acquired immunity on initial symptomatology and severity of malaria attacks Am J Trop Med Hyg 1999 60 410 420 10466970 10.4269/ajtmh.1999.60.410

[B20] Smith T Schellenberg JA Hayes R Attributable fraction estimates and case definitions for malaria in endemic areas Stat Med 1994 13 2345 2358 10.1002/sim.4780132206 7855468

[B21] Smith DL Guerra CA Snow RW Hay SI Standardizing estimates of the *Plasmodium falciparum* parasite rate Malar J 2007 6 131 10.1186/1475-2875-6-131 17894879 PMC2072953

[B22] Betsi N Koua H FouaBi K *Anopheles funestus* (Giles, 1900), la riziculture et le paludisme dans la région forestière ouest de la Côte d’Ivoire Cahiers Agriculture 2003 12 341 346

[B23] Nzeyimana I Henry M-C Dossou-Yovo J Doannio JM Diawara L Carnevale P Epidémiologie du paludisme dans le sud-ouest forestier de la Côte d’Ivoire (région de Taï) Bull Soc Pathol Exot 2002 95 89 94 12145967

[B24] Adja AM N’Goran EK Koudou BG Dia I Kengne P Fontenille D Chandre F Contribution of *Anopheles funestus*, *An. gambiae* and *An. nili* (Diptera: Culicidae) to the perennial malaria transmission in the southern and western forest areas of Côte d’Ivoire Ann Trop Med Parasitol 2011 105 13 24 10.1179/136485910X12851868780388 21294945 PMC4089788

[B25] Smith DL Dushoff J Snow RW Hay SI The entomological inoculation rate and *Plasmodium falciparum* infection in African children Nature 2005 438 492 495 10.1038/nature04024 16306991 PMC3128496

[B26] Rogier C Henry M-C Spiegel A Diagnostic des accès palustres en zone d’endémie: bases théoriques et implications pratiques Med Trop 2001 61 27 46 11584653

[B27] Doolan DL Dobaño C Baird JK Acquired immunity to malaria Clin Microbiol Rev 2009 22 13 36 10.1128/CMR.00025-08 19136431 PMC2620631

[B28] Audibert M Brun J-F Mathonnat J Henry M-C Malaria and agricultural production: Are there bi-directional effects? The case of coffee and cocoa in Côte d’Ivoire Rev Econ Dév 2009 1 107 12621857716

[B29] De Plaen R Geneau R Teuscher T Koutoua A Seka ML Living in the paddies: a social science perspective on how inland valley irrigated rice cultivation affects malaria in Northern Côte d’Ivoire Trop Med Int Health 2003 8 459 470 10.1046/j.1365-3156.2003.01050.x 12753642

[B30] De Plaen R Geneau R Riziculture de bas-fond, autonomie des femmes et paludisme dans le Nord de la Côte d’Ivoire Cah Agricult 2002 11 17 22

[B31] Audibert M Mathonnat J Henry M-C Malaria and property accumulation in rice production systems in the savannah zone of Côte d’Ivoire Trop Med Int Health 2003 8 460 467 10.1046/j.1365-3156.2003.01051.x 12753643

[B32] Audibert M Mathonnat J Nzeyimana I Henry MC Rôle du paludisme dans l’efficience technique des producteurs de coton dans le nord de la Côte d’Ivoire Rev Econ Dév 1999 4 21 14821857716

[B33] Thomson MC D’Alessandro U Bennett S Connor SJ Langerock P Jawara M Todd J Greenwood BM Malaria prevalence is inversely related to vector density in the Gambia, West Africa Trans R Soc Trop Med Hyg 1994 88 638 643 10.1016/0035-9203(94)90204-6 7886754

